# Rheumatic heart disease in pregnancy: a report of 2 cases

**DOI:** 10.11604/pamj.2017.28.298.14159

**Published:** 2017-12-08

**Authors:** Asaph Ziruma, Munyaradzi Innocent Nyakanda, Annie Fungai Muyotcha, Fungisai Nyarai Hove, Mugove Gerald Madziyire

**Affiliations:** 1 Department of Obstetrics and Gynaecology, University of Zimbabwe, College of Health Sciences, Harare, Zimbabwe; 2 Department of Obstetrics and Gynaecology, Parirenyatwa Central Hospital, Harare, Zimbabwe

**Keywords:** Mitral stenosis, mechanical heart valve, pregnancy

## Abstract

Pregnant women with severe mitral stenosis tend to experience clinical decompensation with approximately 50% mortality and they may experience adverse effects of the medication they are taking, notably congenital malformations from warfarin exposure. Corrective heart surgery may increase the risk of pregnancy loss. We present 2 cases of RHD in pregnancy. The first case was a 27-year-old patient in her first pregnancy with severe mitral stenosis. Caesarean section was done for foetal distress and she delivered a small for gestational age baby. She was closely monitored postpartum and was stable on discharge. She presented with supraventricular tachycardia and died in the coronary care unit 4 weeks postpartum. The second case was a 28-year-old who was on warfarin for a mechanical mitral valve. A foetal anomaly scan done at 20 weeks showed severe congenital malformations which were not compatible with extra-uterine life. The pregnancy was terminated and she recovered well. The first case illustrates the significant mortality risk with uncorrected severe rheumatic heart disease. The second case highlights the risks of warfarin on the foetus and the need to avoid mechanical heart valves if possible in young women. RHD patients require preconception counselling so they can make informed reproductive choices.

## Introduction

Cardiovascular disease complicates 1% to 3% of all pregnancies and accounts for 10% to 15% of maternal mortality [1]. In low income countries Rheumatic Heart Disease (RHD) accounts for approximately 90% of all cardiovascular disease among pregnant women [2]. RHD is a disease of the young and its impact is seen in women of reproductive age. For some of these women an initial diagnosis is made antenatal or postpartum, as they fail to tolerate the impact of the physiological changes of pregnancy on their damaged heart valves resulting in clinical decompensation [1]. Symptomatic mitral stenosis (MS) has been associated with higher risk for antenatal hospital admission and approximately 50% mortality, which occurs most commonly in the postpartum period [3]. Though pregnancy for most women is indolent, it presents unique challenges for women with RHD. Besides the impact of the pregnancy and delivery on the heart, considerations also have to be made on the impact of RHD therapy on the foetus. Some medical therapies may be teratogenic, while surgical management in pregnancy may increase the risk of pregnancy loss. We present 2 cases of RHD in pregnancies with adverse maternal and foetal outcomes respectively.

## Patient and observation

**Case 1**: Mrs. A was a 27 year old patient in her first pregnancy who was referred at 15 weeks gestation from a physician with a diagnosis of RHD. She had just been diagnosed with severe MS 3 weeks prior to presentation although she had been symptomatic since childhood. An echocardiogram showed mitral valve area (MVA) of 1cm^2^, Ejection Fraction (EF) of 59.3% and a moderately dilated left atrium of 55-60mm diameter with no pulmonary hypertension. A multidisciplinary team (MDT) was set up comprising a physician, cardiothoracic surgeon, obstetricians and an anaesthetist. The cardiothoracic surgeon offered open commissurotomy as percutaneous balloon valvuloplasty (PBV) was not available. She declined the in view of the procedure related risks. At 20 weeks gestation she was commenced on propranolol to treat a persistent tachycardia and prophylactic warfarin. She was switched from warfarin to low molecular weight heparin (LMWH) at 36 weeks gestation. At 38 weeks, an echocardiogram showed an MVA of 0.9cm^2^ and EF of 58%. Her functional status was New York Heart Association (NYHA) class II. The estimated foetal weight (EFW) was 1950g, with normal middle cerebral artery (MCA) and umbilical artery (UA) flow. In order to time the required lapse period between last dose of LMWH and the epidural procedure, she had elective induction of labour. She developed foetal bradycardia 6 hours after initiating induction. An emergency caesarean section was done under general anaesthesia as the epidural catheter which had been timed for established labour had not yet been inserted. She was given frusemide and antibiotics peri-partum. A 2020g live baby was delivered. She was discharged from hospital on day 7 on warfarin and was stable when reviewed by both physician and obstetrician 14 days postpartum. She was admitted to the Coronary Care Unit 4 weeks postpartum with shortness of breath, severe supraventricular tachycardia and hypotension and died 3 days later.

**Case 2**: Mrs. B is a 28 years old who presented at 10 weeks gestation. She was known to have RHD and was on warfarin for a mechanical mitral valve. The pregnancy had been planned against advice from her gynaecologist and cardiologist. She was asymptomatic (NYHA class I). She was started on folic acid 5mg daily. Her INR was within therapeutic range. At 20 weeks, a foetal anomaly scan showed severely dilated cerebral ventricles dangling choroid plexus, lemon shaped head. The inter-hemispheric fissure was present and there was no identifiable cortical brain tissue. The facial anatomy was normal. There was a discordant growth pattern with the bi-parietal diameter corresponding to 24 weeks, head circumference to 23 weeks but abdominal circumference and femur length both to 19 weeks. A repeat scan by a radiologist showed “evidence of a well formed cranium but with severe hydraencephaly with replacement of supratentorial brain parenchyma by cerebro-spinal fluid. There was mild preservation of posterior fossa structures with ventricles not easily describable.” In the opinion of both the sonographer and the radiologist the findings were not compatible with extra-uterine life. The couple was informed and termination of the pregnancy (TOP) was done. Her INR was 2.3. She expelled the conceptus complete within 24hrs of induction. There was no excessive bleeding and she was discharged in a stable condition. Copper T intrauterine contraceptive device (Cu-IUCD) was offered.

## Discussion

We have presented 2 cases with valvular damage secondary to RHD who complicated with maternal death and congenital malformations respectively. The first case had not received preconception counselling. She was at high risk of poor maternal outcome as predicted by an MVA of 1cm^2^. The second case was high risk because of the mechanical heart valve (MHV) which is highly thrombogenic and requires prophylactic anticoagulation with warfarin. Both patients were managed appropriately by an MDT. Risks and benefits of transcatheter or surgical intervention, including mechanical/bio-prosthesis and valve repair should be discussed with such patients [4]. If possible, cardiac surgery should be avoided during pregnancy. MHVs increase the risk of pregnancy-related thromboembolic complications and the risk of anticoagulant therapy. Bio-prosthetic valves have the challenge of limited durability however women with bio-prosthetic heart valves who are haemodynamically stable tend to tolerate pregnancy very well and do not require anticoagulation. Valve replacement in women of reproductive age should be avoided if other interventions can be instituted. PBV would have been the preferred option because of the relatively low risk of maternal death and foetal loss [4]. The foetus in the second case developed hydraencephaly. Documented features of warfarin embryopathy include microcephaly, hydrocephalus, ventriculomegaly, agenesis of the corpus collosum and Dandy Walker malformation [5]. Hydraencephaly has not been documented in association with warfarin embryopathy, however the full spectrum of warfarin embryopathy is not fully understood and the foetal abnormalities seen in our case could be as a result of warfarin exposure. Warfarin is the most reliable anticoagulant for women with MHVs but is associated with a risk of warfarin embryopathy as well as a substantially increased risk of foetal loss [6]. Warfarin crosses the placenta and causes haemorrhage in foetal organs. The period of greatest risk of embryopathy is between the 6^th^ and 9^th^weeks of gestation resulting in skeletal and facial anomalies. Exposure in the second and third trimesters may result in central nervous system abnormalities [7]. On the other hand, unfractionated heparin and LMWH are associated with an increased incidence of thromboembolism in patients with MHVs [6]. Women on warfarin are considered to be at risk of having a foetus with neural tube defects (NTD) and should be on 5mg of Folate supplementation. Supplementation should be started preconception [8]. The second case was unfortunately started on folate at 10 weeks gestation which was her first visit.

The first case was put on propranolol to control persistent tachycardia. Arrhythmias such as atrial fibrillation and ventricular tachycardia are common complications in patients with RHD. The atrial and ventricular rate and rhythm can be controlled by using selective oral beta-blockers, calcium channel blockers, amiodarone or digoxin [9]. Although a systematic review could not make conclusions on the effect of beta-blockers like propranolol on perinatal mortality and preterm birth, there was sufficient evidence that they do increase neonatal bradycardia and small for gestational age infants, apnoea, hypoglycaemia and hyperbilirubinemia [10]. The baby had low birth weight which might be attributed to intrauterine propranolol exposure. Non-selective beta-blockers like labetalol have fewer documented adverse foetal outcomes, but do not have proven efficacy in the treatment of arrhythmias. Amiodarone use in pregnancy has been associated with transient hypothyroidism, goitre and mild neurodevelopmental abnormalities in the foetus or new born. It is only indicated where maternal tachyarrhythmia is refractory to other safer drugs [11]. Digoxin easily crosses the placental barrier and is associated with increased risk for miscarriage and foetal death when the mother is taking very high doses [12]. The 1^st^ case decompensated a month after delivery and died under physician care. The risk of maternal mortality is highest in the third trimester and puerperium [3]. Immediately postpartum, the cardiac output remains elevated in comparison to antenatal values due to the relief of caval compression, auto-transfusion from the uterus and continued resorption of extracellular fluid into the intravascular compartment. High level surveillance is required in the postpartum period until the haemodynamic changes have resolved. For unstable patients, hospital monitoring might be required for an extended period. Women with RHD particularly need to have planned pregnancies. Women taking anticoagulants, need a reliable contraceptive method which will not increase thrombotic risk, reduce menstrual blood loss and inhibits ovulation. The choice of contraceptive method should be based on the impact that an unplanned pregnancy will have, risks and benefits of each type and individual preferences. Sterilization is an option for the woman in the second case but she got the Copper T intra-uterine contraceptive device (Cu-IUCD) because it is difficult for women who have never conceived to accept permanent methods. Combined oral contraceptives are contraindicated in women with a history of mechanical heart valves [13].

## Conclusion

Women with RHD of reproductive age must receive early preconception evaluation and advice regarding the potential impact of pregnancy on their cardiovascular function. Those who chose to conceive or present after conception need management by a MDT with emphasis on identifying and avoiding triggers of decompensation and fetal anomaly/loss throughout pregnancy and the puerperium.

## Competing interests

The authors declare no competing interests.
